# Efficacy of a novel drainage catheter in the treatment of CSF leak after posterior spine surgery: A retrospective cohort study

**DOI:** 10.1515/med-2025-1211

**Published:** 2025-05-29

**Authors:** Shu Yang, Zhengyuan Zhu, Gang Chen, Zhisheng Long

**Affiliations:** Department of Orthopedics, Jiangxi Provincial People’s Hospital, The First Affiliated Hospital of Nanchang Medical College, Nanchang, Jiangxi, 330006, China; Medical College, Nanchang University, Nanchang, Jiangxi, 330006, China; Department of Orthopedics, DeXing People’s Hospital, DeXing, Jiangxi, 334200, China

**Keywords:** cerebrospinal fluid leak, posterior spinal surgery, cerebrospinal fluid drainage, surgical complications

## Abstract

**Purpose:**

This work aimed to evaluate and analyze the efficacy and safety of modified drainage and catheterization in the treatment of cerebrospinal fluid leakage (CSFL) after posterior spinal surgery.

**Methods:**

A total of 12 patients with CSFL were selected from June 2020 to June 2023. The modified drainage technique was used to treat CSFL. The perioperative index, including the duration of drainage tube placement, was observed. The study also assessed the amount of cerebrospinal fluid (CSF) drainage, the healing time of the drainage wound, the occurrence of other complications, the effectiveness of the drainage procedure, and the length of hospital stay.

**Results:**

Among the patients with CSFL, the mean drainage time was 6.2 ± 0.8 days, the healing time of the drainage wound was 9.6 ± 1.7 days, and the hospital stay was 7.5 ± 1.0 days. The total volume of CSF drainage was 488.6 ± 55.3 mL. Furthermore, no cases of retrograde infection, drainage orifice exudate, or drainage wound nonunion were reported in this study.

**Conclusion:**

This preliminary study demonstrates that the modified catheterization technique is effective in reducing the duration of CSFL and promoting rapid wound healing. Additionally, the novel drainage catheter shows advantages in terms of reducing the incidence of infection and other related complications.

## 1 Introduction

Cerebrospinal fluid leakage (CSFL) is a common complication observed in posterior spinal surgery [1,2]. It occurs as a result of intraoperative dura mater injury, which leads to CSFL. This can be identified through postoperative incisions or drainage fluid [3,4]. The incidence of CSFL caused by intraoperative dural injury is increasing due to the growing number of patients with peridural adhesion, dural thinning, and those undergoing secondary surgery. In the clinical management of posterior spinal surgery, the occurrence of CSFL is approximately 14% in posterior lumbar surgery and around 1% in posterior cervical surgery [5].

CSFL is frequently encountered in spinal surgery, with many patients experiencing symptoms such as headache, vomiting, nausea, hearing loss, facial numbness, dizziness, photophobia, and signs of central nervous system infection (including meningoencephalitis and brain abscess) [5,6]. CSFL can lead to various complications, including infectious complications (such as meningitis, arachnoiditis, and wound infection), delayed wound healing, complications of intracranial hypotension (such as intracranial hemorrhage and cranial nerve palsy), as well as neurological disorders resulting from nerve compression or entrapment [7]. In cases of CSFL following posterior spinal surgery, drainage is often employed. Common methods of drainage after posterior spinal surgery include continuous drainage in the operative area, lumbar cistern drainage [3,8,9], or subarachnoid drainage [10]. However, these drainage methods can create a straight sinus at the fixed site, involving the fascia, muscle, and subcutaneous tissue, which may hinder normal tissue involution and subsequently delay wound healing. This, in turn, increases the risk of meningitis caused by retrograde infection. Recently, researchers have proposed using long-term subfascial and epidural drainage as an alternative to lumbar subarachnoid drainage [2–5]. This approach allows sufficient time for the healing of the lumbar dorsal fascia and skin before removing the drainage tube. It is believed that after the tube is removed, the pressures in the epidural and subarachnoid spaces balance out, preventing cerebrospinal fluid (CSF) from flowing through the dural defect and promoting its healing [10]. However, there is currently a lack of high-level research to definitively prove the effectiveness of subfascial drainage in reducing the risk of retrograde CSF infection. The modified drainage and catheterization technique, inspired by abdominal puncture technology, incorporates a Z-shaped puncture combined with subfascial drainage for the treatment of patients with CSFL following posterior spinal surgery.

The aim of this study is to assess the therapeutic potential, efficacy, and safety of the modified drainage technique in patients with CSFL following posterior spinal surgery. The findings of this study can serve as a reference for future clinical applications.

## 2 Subjects and methods

### 2.1 Study design

This study was approved by the Ethics Review Committee of Jiangxi Provincial People’s Hospital, and all patients signed informed consent to participate in this study. This retrospective cohort study (without control group) included 12 patients (7 males and 5 females) complicated with CSFL after posterior spinal surgery in our hospital from June 2020 to June 2023. Twelve patients with CSFL were treated with modified drainage and epidural subfascial drainage.

The inclusion criteria for this study were as follows:

(1) Individuals diagnosed with primary diseases such as idiopathic scoliosis, secondary scoliosis, intraspinal tumor, ossification of ligamentum flavum, or lumbar spinal stenosis. All primary diseases were complicated by CSFL due to dura mater damage caused by accidents or self-disease after posterior spinal surgery. Patients who underwent direct suture operation, fascial patch repair, artificial spinal patch repair, or other meningeal repair treatments were included.
(2) All patients who voluntarily participated in the study and signed the informed consent form.

The exclusion criteria were as follows:

(1) CSFL due to dura mater damage caused by compression and infection in the primary disease;
(2) patients with excessive dura mater injury after surgery without effective dural repair;
(3) complicated with severe dysfunction of other major organs;
(4) cases with severe infection or malignant tumor;
(5) cases with diabetes, malnutrition, cancer, or infectious diseases of the spine such as suppurative spondylitis; and
(6) the above information is incomplete.

These criteria were adapted from previous studies [9,12]. These inclusion and exclusion criteria were partially modified based on our research objectives as follows: (1) all our surgeries are posterior approaches; therefore, inclusion criteria included procedures such as posterior scoliosis correction and posterior lumbar decompression. (2) Exclusion criteria included conditions like tumors, infections, and underlying diseases such as diabetes and malnutrition, which could affect the evaluation of final outcomes.

### 2.2 Method of catheter drainage

**Figure 1:**
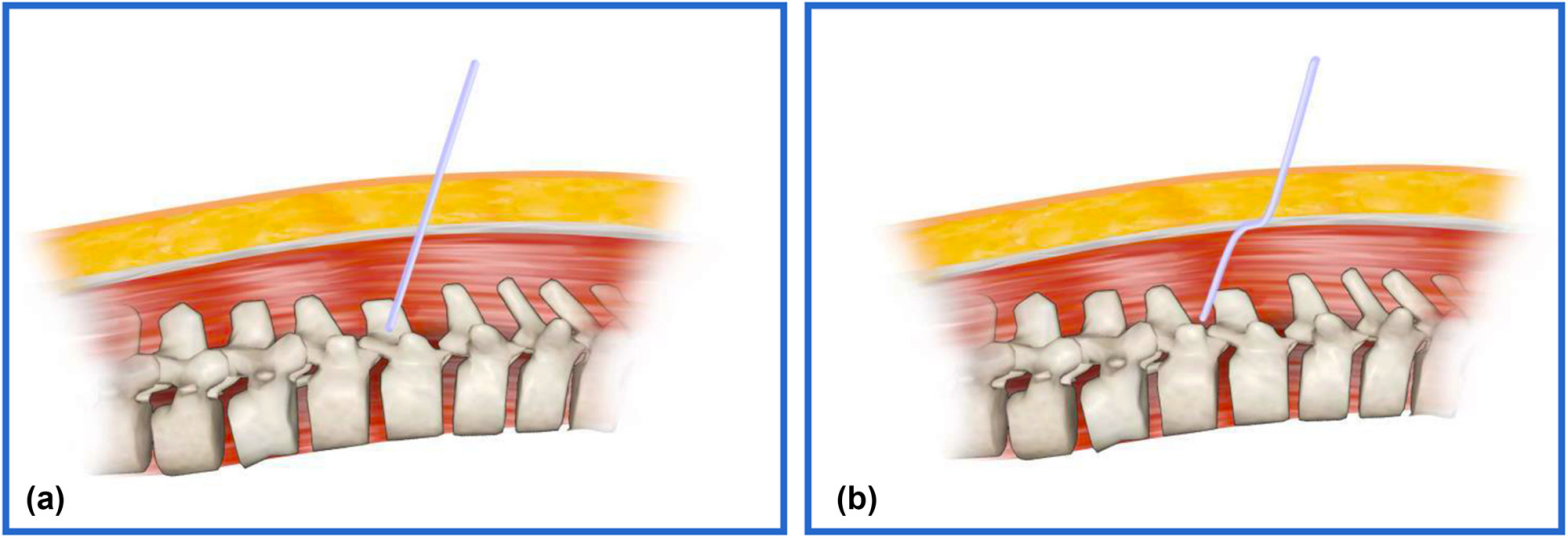
(a) Traditional method of drainage puncture and placement of cerebrospinal fluid leaks involves the puncture needle passing straight through the skin, fascia, and muscle, with the drainage tube forming a straight line between the skin, fascia, and muscle layers. (b) Modified drainage puncture and placement technique shows a 45° oblique puncture to the skin to reach the surface of the lumbar dorsal fascia; it then moves 1.5–2 cm longitudinally across the surface of the lumbar dorsal fascia and finally 45° obliquely out of the lumbar dorsal muscle layer. The lumbar muscle layer is then passed diagonally at 45° until it reaches the peri-vertebral plate. This results in a zigzag pattern of drainage tunneling through the subcutaneous, fascial, and muscular layers.

The method of catheter drainage was used to address the patient’s CSFL during surgery. The modified technique involved the following steps: (1) A needle was inserted through the skin at a 45° angle to reach the lumbar dorsal fascia. (2) The needle was then moved longitudinally 1.5–2 cm over the lumbar dorsal fascia and finally diagonally at 45° through the lumbar musculature to reach the perivertebral plate. This created a drainage tunnel that was misaligned between the fascia and the musculature. The key aspect of the modified drainage tube operation was adjusting the angle of the puncture needle twice in the lumbar dorsal fascia and the muscle layer of the lower back. Additionally, the longitudinal needle had to penetrate a certain distance (Figure 1).

### 2.3 Management of the perioperative period

After the diagnosis of CSFL was confirmed, all patients were prescribed concomitant acetazolamide at a dosage of 250 mg three times a day [11–13]. Intravenous administration of broad-spectrum antibiotics was initiated to control the infection. Patients were advised to rest in bed in the semi-Fowler position, and pressure dressings were applied around the surgical incision [3]. Throughout the treatment, careful monitoring of drainage was conducted, and measures were taken to prevent additional CSFL by avoiding symptoms such as coughing, urinary retention, and nervousness.

### 2.4 Statistical analysis

The data (demographic and clinical data of included patients and clinical data) in this current study were exhibited as mean ± standard deviation (SD) and were analyzed by SPSS Statistics for Windows, Version 23.0.(IBM Corp. Armonk, New York, USA).


**Informed consent:** Informed written consent was obtained from the patient to publish their personal or clinical details information.
**Ethical approval:** The Ethical Committee of Jiangxi Provincial People’s Hospital approved this study (No. KT019). All clinical investigations were conducted following the principles expressed in the Declaration of Helsinki. All participating work units have passed ethical approval for this study, approved the use of case data, and authorized this study.

## 3 Results

### 3.1 Demographic and clinical data

The demographic and clinical data of included 12 patients (7 males and 5 females). The mean age of patients was 56.6 ± 22.1 years. The preoperative diagnoses were scoliosis (three cases), tumors in the spinal canal (three cases), ossification of the ligamentum flavum (three cases), and lumbar spinal stenosis (two cases) (Table 1).

**Table 1:** Demographics of the patients preoperatively as well as perioperative data and complications after drainage

Age	Gender	Preoperative diagnosis	Term of operation	Curative effect	Complications after drainage	Drain duration day (days)	Drained volume (mL)	Hospital stay (days)	MWHT (days)
13	Female	Scoliosis	Vertebrae osteotomy and thoracolumbar fusion	Cured	—	6	566	8	13
13	Female	Scoliosis	Vertebrae osteotomy and thoracolumbar fusion and dural suture	Cured	—	6	489	9	10
64	Male	Scoliosis	Vertebrae osteotomy and thoracolumbar fusion	Cured	—	7	455	8	10
70	Female	Scoliosis	Vertebrae osteotomy and thoracolumbar fusion	Cured	—	6	492	8	12
74	Male	Intraspinal tumor	Excision of intraspinal tumor and dural suture	Cured	—	6	562	7	9
43	Male	Intraspinal tumor	Excision of intraspinal tumor	Cured	—	5	402	7	10
66	Female	Intraspinal tumor	Excision of intraspinal tumor	Cured	—	7	508	9	11
72	Male	OLF	Local laminectomy and flavectomy	Cured	—	6	522	6	8
63	Male	OLF	Local laminectomy and flavectomy	Cured	—	6	531	7	9
73	Female	OLF	Local laminectomy and flavectomy	Cured	—	5	488	8	9
72	Male	SCS	Endoscopic extraction of lumbar nucleus pulposus	Cured	—	7	392	6	7
56	Male	SCS	Endoscopic extraction of lumbar nucleus pulposus	Cured	—	8	456	7	8

MWHT: mean wound healing time; OLF: ossification of the ligament flavum; SCS: spinal canal stenosis.

### 3.2 Analysis of perioperative indicators in patients with CSFL

The days of drainage tube placement after the improved drainage tube technique combined with epidural subfascial drainage in patients with CSFL were 6.2 ± 0.8 days, the incision healing time was 9.6 ± 1.7 days, the hospital stay was 7.5 ± 1.0 days, the mean total CSF drainage was 488.6 ± 55.3 mL. Perioperative parameters are shown in Table 1.

### 3.3 Recovery rate and complications of CSFL after drainage

After adopting the improved drainage technique combined with epidural subfascial drainage for CSF drainage, all 12 CSF patients were cured and no CSFL, serious complications such as pseudodural cyst, skin sinus or fistula formation, retrograde infection, etc., occurred. The clinical data of cure and CSFL complications are shown in Table 1.

### 3.4 Typical case

**Figure 2:**
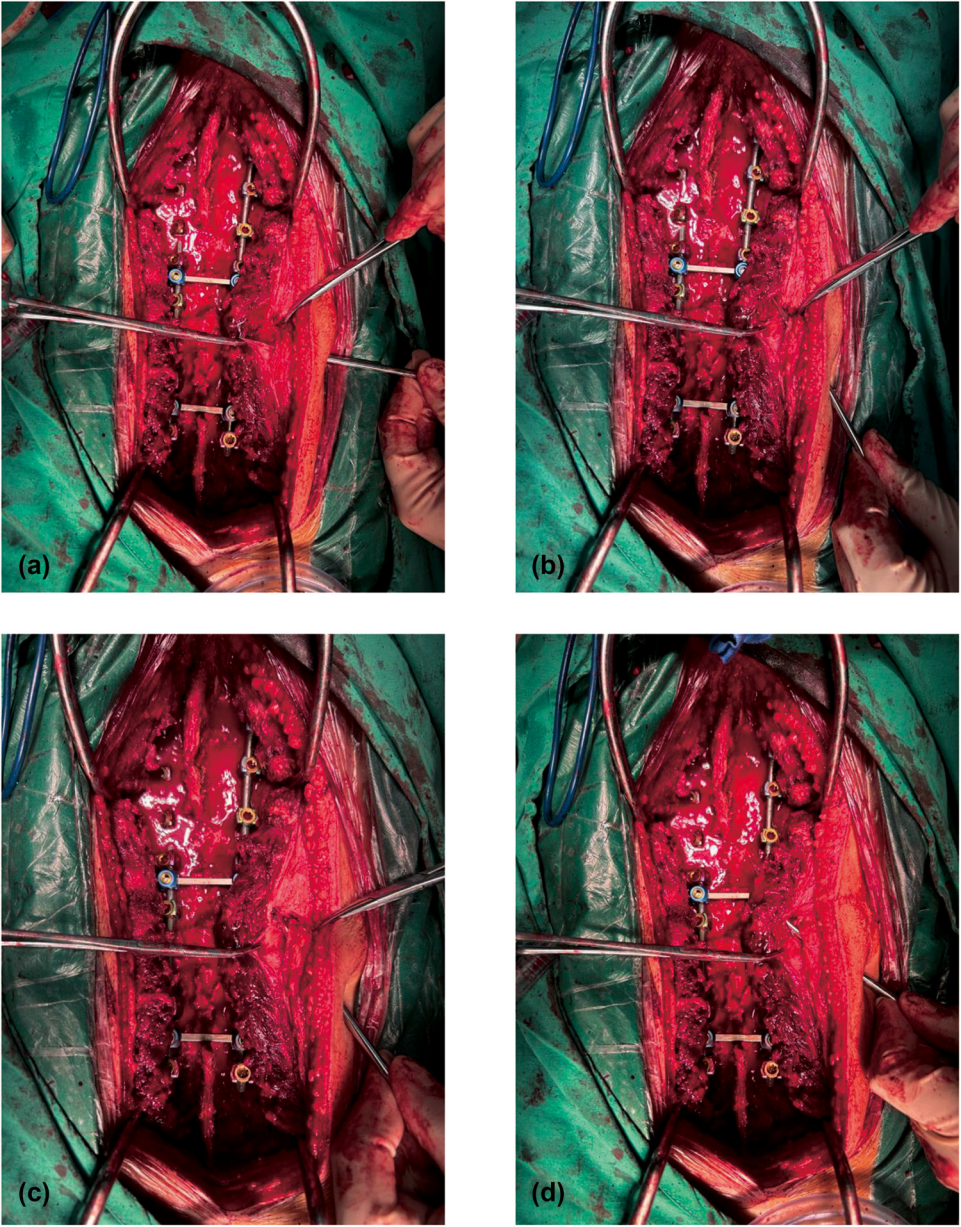
Modified drainage catheter for CSFL during scoliosis surgery. (a) Puncture the needle into the skin and subcutaneous tissue at a 45° angle. (b) The puncture needle was placed 1–2 cm parallel to the surface of the dorsal fascia of the lumbar spine. (c) The needle is inserted into the muscles of the lower back at an angle of 45°, so that the tunnels in the muscles and subcutaneous tissue are arranged in a Z-shape. (d) In the end, the drainage system passes through tunnels in two different levels.

The 13-year-old female patient was diagnosed with scoliosis and had previously undergone scoliosis correction surgery at the age of one. The specific diagnosis was juvenile idiopathic scoliosis. During the operation, it was discovered that the patient had experienced a CSFL. To address this, a modified catheter drainage method was employed, with the drainage tube placed in a Z-shape between the muscle and subcutaneous area. The drainage tube remained in place for 6 days, and the patient stayed in the hospital for a total of 9 days. The drainage incision was closed on the 13th day after the operation. A total of 489 mL of CSF was drained. There were no noticeable complications during the follow-up examinations at 1, 3, 6, and 12 months (Figure 2).

## 4 Discussion

CSFL after posterior spinal surgery is often caused by inadvertent injury to the dura mater during surgery. This can result in CSFL from the incision or drainage fluid after the surgery. The incidence of CSFL due to intraoperative dural damage increases with factors such as the presence of peridural tissue adhesions, thin dura mater, and secondary surgeries. Even experienced surgeons may not always detect small dural incisions during the operation, leading to postoperative CSFL in some patients. According to Mammadkhanli et al., the incidence of unintentional durotomy in spinal surgery ranges from 1 to 17% [3]. Postoperative CSFL can give rise to various complications, including infectious complications (such as meningitis, arachnoiditis, and wound infection), delayed wound healing, complications related to intracranial hypotension (such as intracranial hemorrhage), and nerve dysfunctions caused by nerve compression or incarceration.

In order to prevent and manage CSFL after posterior spinal surgery, appropriate prevention and drainage methods were implemented. One common method is careful intraoperative suturing of the fascia and muscle, or patch suturing to repair any damage to the dura mater [14]. However, a study by Jankowitz found that using fibrin glue to repair the dura mater did not significantly reduce the incidence of persistent CSFLs [15]. Another study by Agak utilized negative pressure wounds to treat CSFLs, but this approach can potentially lead to brain herniation due to excessive drainage of CSF [16]. Additionally, Xianda et al. reported that fat grafting combined with paraspinal muscle flap can be used to treat CSFLs after thoracolumbar posterior approach surgery, but there is currently limited high-level evidence to support its effectiveness [17]. Three methods are currently used to drain CSFLs: continuous surgical drainage, lumbar subarachnoid drainage, or lumbar cistern drainage. However, these drainage methods can create a direct pathway for infection at the fixation site, fascia, muscle, and subcutaneous tissue. This increases the risk of meningitis caused by retrograde infection. Hussein et al. reported a success rate of subarachnoid drainage between 85 and 94%, but the complication rate can be as high as 44% [9]. The most serious complications include brain herniation due to excessive drainage, symptoms of low intracranial pressure, hydrocephalus, and meningeal infection. Recent research by Fang indicated that postoperative CSFL can be prevented by reducing CSF pressure and increasing epidural space pressure. This helps delay CSF flow and promote the repair of damaged dura mater [2,3,11]. Therefore, subfascial drainage is often used to eliminate the dead space. The combination of subfascial drainage and the clipping technique can effectively balance the pressure gradients and promote the formation of granulation tissue [3,11]. However, retrograde infection through the drainage port remains a concern.

Recent literature has highlighted the ongoing challenges in managing CSFLs after spinal surgery, with a focus on minimizing complications and improving patient outcomes [18,19]. Various techniques, including the use of novel biomaterials for dural repair and advanced wound closure methods, have been explored to reduce the incidence of persistent CSFL [20,21]. However, despite these advances, the risk of retrograde infection and delayed wound healing remains a significant concern [22]. Our modified drainage technique, characterized by a Z-shaped subfascial tunnel, offers several distinct advantages over traditional straight-line drainage methods. This is consistent with recent findings that innovative drainage and closure strategies can significantly decrease infection rates and promote faster wound healing [23,24].

In this pilot study, we treated CSFL after posterior spinal surgery using subfascial drainage with a modified catheterization technique. The drainage tube was in place for an average of 6.2 ± 0.8 days, incision-healing time was 9.6 ± 1.7 days, hospitalization time was 7.5 ± 1.0 days, and the total volume of CSF drained was 488.6 ± 55.3 mL. Our results showed that our modified drainage catheterization technique had advantages over previous studies in terms of shorter drainage tube placement, faster wound healing, and reduced hospital stay compared to studies by Tang [9] and Mammadkhanli [3] who used simple subfascial epidural drainage (Table 2).

**Table 2:** Perioperative data and incidence of complications

Parameter	Current study	Tang et al. [9]	Mammadkhanli et al. [3]
Drain duration day (days)	6.2 ± 0.8	7.1 ± 0.5	7.4 ± 1.0
Hospital stay (days)	7.5 ± 1.0	13.1 ± 2.7	8.4 ± 1.0
Infection rate (%)	0	0	0

In this preliminary study, “complication-free was defined as the absence of retrograde infection, pseudodural cyst formation, wound nonunion, or other adverse events. These outcomes were systematically assessed through regular follow-up and medical record review.” No major drainage-related complications, such as unreduced CSFL, pseudodural bulge, skin sinus or fistula formation, and retrograde infection, were observed in the enhanced catheterization group.

These findings suggest that the use of enhanced catheterization tube technology may help reduce the risk of post-drainage complications, particularly retrograde infection, and consequently decrease the length of hospital stay for patients. From our perspective, this novel catheterization technology has several advantages. First, the modified drainage tube placement technique creates a Z-shaped staggered layer in the subcutaneous, lumbar dorsal fascia, and lumbar dorsal muscles. This technique effectively reduces the risk of retrograde infection through the drainage orifice and slows down the drainage speed of CSF by increasing the resistance of CSFL and exudation. As a result, it alleviates the symptoms of low intracranial pressure caused by rapid drainage, leading to improved clinical prognosis and shorter hospital stays for patients. Second, the deep lumbar fascia, known for its high tensile strength among closed tissues, provides significant resistance to CSF flow. By forming an approximately Z-shaped staggered layer tunnel in combination with epidural drainage, early healing of the deep lumbar fascia is effectively promoted. Furthermore, after the removal of the drainage tube, the pressure beneath the fascia and in the intrathecal space tends to equalize, indirectly slowing down the CSFL post-removal.

## 5 Limitations

However, it is important to acknowledge the limitations of this study. First, as a retrospective study, it relies on medical records for data collection, which may introduce information bias. Second, the lack of a control group limits the ability to directly compare the modified drainage technique with conventional methods. Third, the sample size is relatively small, and the follow-up period is short, which may affect the generalizability of the findings. Future prospective, randomized controlled trials with larger sample sizes and longer follow-up periods are necessary to validate these preliminary results and provide higher-level evidence.

## 6 Conclusion

Our current study has preliminarily revealed that modified drainage tube placement technology can significantly reduce the duration of CSFL and promote rapid wound healing after surgery. This technology is expected to become a novel and potential treatment method for CSFL in the future.

## Abbreviations

CSF: cerebrospinal fluid
CSFL: cerebrospinal fluid leak
